# Inflammatory Effects of the Plant Protection Product Stifenia (FEN560) on Vertebrates

**DOI:** 10.3389/fpubh.2017.00074

**Published:** 2017-04-24

**Authors:** Lény Teyssier, Julie Colussi, Stéphanie Delemasure, Johanna Chluba, David Wendehenne, Olivier Lamotte, Jean-Louis Connat

**Affiliations:** ^1^Univ. Bourgogne Franche-Comté, LNC UMR866, Dijon, France; ^2^UMR1347 Agroécologie, AgroSup Dijon, INRA, Univ. Bourgogne Franche-Comté, Dijon, France; ^3^ERL CNRS 6003, Dijon, France; ^4^Cohiro Biotechnology, Faculté de médecine, Dijon, France

**Keywords:** peripheral blood mononuclear cells, zebrafish, IL-1β, pesticides, plant defense stimulator, fenugreek

## Abstract

Plant defense stimulators (PDSs) rely on the activation of plant innate immunity in order to protect crops against various pests. These molecules are thought to be a safer alternative to classical plant protection products. Given that innate immune systems share common features in plants and vertebrates, PDS can potentially cross-react with innate immunity of non-target organisms. To test this hypothesis, we studied effects of the commercial PDS Stifenia (FEN560), which is composed of crushed fenugreek seeds. We tested various concentrations of Stifenia (0.03–1 mg mL^−1^) on human peripheral blood mononuclear cells and checked, 20 h later, cell metabolic activity (MA) using XTT assay, cell death by flow cytometry analysis, and IL-1β inflammatory cytokine released in the culture medium using ELISA. Stifenia induced a general decrease of the cell MA, which was concomitant with a dose-dependent release of IL-1β. Our results highlight the activation of human immune cells. The inflammatory effect of Stifenia was partially inhibited by pan-caspase inhibitor. Accordingly, Stifenia induced the release of p20 caspase-1 fragment into the culture medium suggesting the involvement of the NLRP3 inflammasome. Furthermore, we observed that Stifenia can induce cell death. We also tested the effect of Stifenia on Zebrafish larvae. After 24 h of exposure, Stifenia induced a dose-dependent IL-1β and TNFα gene expression. The human-cell-based approach developed in this work revealed a high sensitivity concerning inflammatory properties of a plant protection product. These tests could be routinely used to screen the potential adverse effects of this type of compounds. Finally, our results suggest a potential danger of using extensively certain PDS for crop protection.

## Introduction

In the context of pesticides reduction, alternative strategies to protect crops have emerged, including use of transgenic crops, resistant hybrids, or integrated pest management methods. Among these, stimulation of the plant immune system with various molecules is promising. Plant defense stimulators (PDSs), plant defense inducers, or elicitors define a class of compounds of diverse origins, which can induce disease resistance-related mechanisms by mimicking a pathogen attack or a danger state, resulting in reduced levels of plant infection. They comprise a range of purified or mixture-based natural or synthetic compounds that have been shown to protect plants efficiently (1, 2). To use a new molecule for crop protection in France, an authorization is needed according to the European Union (EC No. 1107/2009) and French regulations. Various toxicological and ecotoxicological tests are required for the production of an initial draft assessment report by EU-designated rapporteur member state (RMN) (3). Stifenia (FEN560), which is exclusively composed of grounded fenugreek seeds (*Trigonella foenum-graecum*) is a PDS authorized by the French Agency for Food, Environmental and Occupational Health and Safety (ANSES) to fight powdery mildew of grape vine (*Erysiphe necator*) and powdery mildew of melon (*Podosphaera fuliginea and Golovinomyces cichoracearum*) (ANSES agreement no. 2012-1685 and 2013-0227). Fenugreek, especially its seeds and its leaves, has been used for centuries in India and North Africa as food or in traditional medicine (4).

Similar to animals, the first step in the activation of the plant immune system is the perception of pathogens or microbe-associated molecular patterns (PAMPs/MAMPs) by pattern recognition receptors (5, 6). In both organisms, their perception induces complex cell signaling events, which result in cellular re-programming. For instance, PAMP/MAMP-triggered immunity in plants is associated with the production of phytoalexins, a class of antimicrobial metabolites, and with the reinforcement of plant cell walls. In mammals, the immune response induces, for example, the production of cytokines (interleukines, TNFα) and antimicrobial peptides. One of the inflammatory cytokine that plays a major role is IL-1β. Indeed, this cytokine is produced following inflammasome activation in monocytes, macrophages, and dendritic cells upon their stimulation by PAMP or damage/danger-associated molecular patterns. IL-1β is processed from pro-IL1β by caspase1 in several inflammasome complexes (e.g., NLRP3, NLRP1, and AIM2) (7, 8). This cytokine upregulates many other inflammatory factors such as IL6 (9) or TNFα (10).

Since plant and animal immune systems share some similarities (11, 12), we hypothesized that the PDS Stifenia could cross-react with the animal immune system. In fact, both beneficial and adverse effects have been described for fenugreek in mammals (4, 13–18) and toxicity against insects has also been reported (19). We thus tested the effects of Stifenia on human peripheral blood mononuclear cells (PBMCs) from different blood donors by quantifying the amount of the inflammatory cytokine IL-1β released by exposed cells. In parallel, we evaluated the metabolic activity (MA) of the stimulated cells using a XTT assay. We also checked the intensity of cell death induced by Stifenia. Finally, we studied the effects of this compound on the larvae of the model fish zebrafish by analyzing cytokine gene induction.

## Materials and Methods

### Chemicals

Stifenia (FEN560, Société Occitane de Fabrications et de Technologies, France) was extemporaneously suspended in Roswell Park Memorial Institute medium (RPMI; for cell treatment) at 17 mg mL^−1^ or in autoclaved mineral Volvic water (for zebrafish treatment) at 0.1 mg mL^−1^ and gently shaken for 30 min. Since Stifenia is not fully soluble in water, insoluble matters were isolated by centrifugation (20,000 *g*, 30 min, room temperature) and supernatant was carefully collected in a new tube. All the concentrations indicated in this work refer to the initial concentration (17 mg mL^−1^ for human experimentation and 0.1 mg mL^−1^ for zebrafish experimentation).

Z-VAD-FMK stock solution [20 mM in 100% dimethylsulfoxide (DMSO)], purchased from Promega, was first diluted in RPMI at 85 µM. This solution was used for cell treatment. The final Z-VAD-FMK concentration was 5 µM in cell culture (0.025% DMSO). LPS from *Escherichia coli* 0111:B4 stock solution (1 mg mL^−1^ in pure water), purchased from Sigma-Aldrich, was diluted in RPMI to reach a final concentration of 10 ng mL^−1^ in cell culture. TNBS (2,4,6-trinitrobenzenesulfonic acid) stock solution (1 mg mL^−1^ in pure water), purchased from Sigma-Aldrich, was diluted in autoclaved mineral water (Volvic, France) at 75 µg mL^−1^ for zebrafish treatment.

### Human PBMCs

Buffy coats from healthy donors were obtained from EFS Besançon, France (Agreement No. DECO-14-0124). PBMCs were prepared using Pancoll (density 1.077 g mL^−1^, PAN-biotech Gmbh, Germany) and Blood Sep Filter tubes (Dominique Dutscher, France). Briefly, 15 mL of Pancoll were collected into the lower part of a Blood Sep Filter tube by a short centrifugation. Then, 25 mL of buffy coat and 15 mL of Dulbecco’s phosphate-buffered saline (DPBS, PAN-biotech Gmbh, Germany) were added, gently mixed, and centrifuged (400 *g*, 30 min, room temperature) without brake for the deceleration phase. The PBMC ring was collected, washed three times in DPBS without Ca^2+^ and Mg^2+^, and centrifuged (300 *g*, 10 min, 4°C). Cells were suspended in 2–5 mL of DPBS depending on the size of the cell pellet and kept on ice. Viable PBMCs were counted using trypan blue (20), suspended in RPMI medium supplemented with 10% (bovine serum albumin, w/v) and 1% PSA (Penicilline 10,000 U mL^−1^, Streptomycine 10 mg mL^–1^, Amphotericin B 25 µg mL^–1^ prepared in water), and then seeded in 96-well plate with 10^5^ cells per well in 150 µL of medium.

Immediately after seeding, treatments were done by adding 10 µL per well of a 17-fold concentrated solution of Stifenia made by serial dilution from the stock solution described above. For anti-inflammatory studies, 30 min after Stifenia exposure, 10 µL of LPS solution was added to reach a final concentration of 10 ng mL^–1^. Z-VAD-FMK was added simultaneously to Stifenia treatment. The final volume of RPMI was adjusted to 170 µL for all conditions. Twenty hours after treatment, cells were centrifuged (600 *g*, 3 min). Supernatants were collected for IL-1β quantification, and cell pellets were used for measuring cell MA.

### Cell MA

Cell MA, reflecting cell viability, was determined using the XTT [2,3-bis-(2-methoxy-4-nitro-5-sulfophenyl)-2H-tetrazolium-5-carboxanilide] assay (XTT sodium salt, Sigma-Aldrich, France), based on the reduction of a tetrazolium salt (XTT) by dehydrogenases of viable cells into a water-soluble orange formazan product. Briefly, PBMCs were centrifuged (600 *g*, 3 min) to remove culture medium. Then, 100 µL of RPMI without phenol red and 20 µL of a mixture of 0.9 mg mL^−1^ XTT and 0.01 mM phenazine methosulfate (Sigma-Aldrich, France) were added on pelleted cells. Cells were incubated at 37°C for 4 h and absorbance at 490 nm was measured with a background subtraction at 660 nm using a microplate reader (Infinites M200 PRO, Tecan, France). The results are expressed as percentage of MA compared to control non-exposed cells.

### Quantification of Inflammatory Cytokines

Production of the inflammatory cytokine IL-1β was estimated in the culture medium using ELISA assay (Human IL-1β ELISA Ready-SET-Go! eBiosciences, France) according to the supplier’s instructions.

### Quantification of Cell Death

Two hundred thousand cells were seeded in 24-well plates and treated with Stifenia during 20 h as described above. Cells contained in the culture medium were harvested by centrifugation (400 *g*, 5 min) and then suspended in 300 µL of PBS containing propidium iodide (PI) (10 µg mL^−1^). PI-stained cells were detected using a LSR II flow cytometer (BD Biosciences) and acquisitions were performed during 45 s using BD FACSDiva Software 6.1.2. Flow Jaw was used for figure drawing.

### p20-Caspase-1 Assay

Quantity of processed caspase-1 was evaluated by measuring the amount of the p20 fragment secreted in the culture medium using ELISA assay (Human Caspase-1/ICE Quantikine ELISA Kit, R&D Systems, France) according to the supplier’s instructions.

### Zebrafish Strains, Maintenance, and Treatment

According to the European Union Directive 2010/63/EU, no specific ethics approval was required for this project, as all zebrafish larvae used in this study were less than 120-h postfertilization (hpf) old (21, 22). Wild-type fishes (WIK strain) were obtained from the ZIRC (OR, USA) and kept at 28°C with a light:dark cycle 14:10 h. They were fed twice a day with dried flake food (Gemma Mirco, Skretting, France). The fish were mated and spawning was stimulated by the onset of light. Zebrafish eggs were collected immediately after being fertilized and distributed in 24-well plates (three eggs per well) containing 1 mL of autoclaved mineral water (Volvic, France). At 4 days postfertilization, water was replaced by fresh water containing the desired concentration of Stifenia or 75 µg mL^−1^ of TNBS. Twelve to fifteen zebrafish larvae per condition were incubated during 20 h.

### RNA Extraction and RT-qPCR

After 20 h of exposure (Stifenia or TNBS, see above), 12–15 zebrafish larvae (120 h postfertilization) were euthanized with tricaïne, collected, and disaggregated during 5 min at room temperature using 17-gage needles in 1 mL of Trizol reagent (Invitrogen, France) and vortexed. Total RNAs were extracted according to supplier’s instructions. The RNA samples were treated with DNAse (TURBO DNA-free, Life Technologies) according to the manufacturer’s instructions. Approximatively 15–20 µg of RNA diluted in water were washed with butanol and diethyl ether according to Krebs et al. (23). Diethyl ether was evaporated under a fume hood. The resultant water phase containing RNA was mixed with 175 µL of RNA Lysis Buffer and 350 µL of RNA Dilution Buffer and loaded on a SV Total RNA column (Promega, France) to perform on-column DNAse digestion according to supplier’s instruction. Then, 1 µg of total RNA was reversed-transcribed to cDNA using the iScript™ reverse transcription supermix for RT-qPCR (Biorad, France). Analyses were performed on a thermocycler (Step One Plus, Applied Biosystems, France) using Power SYBR Green from the same purchaser. The parameters used for the PCR were 95°C for 10 min, 40 cycles of 95°C for 15 s, and 60°C for 1 min. The relative expression ratio (experimental/control) was normalized with *Danio rerio* HPRT1 (24) according to 2^−ΔΔCt^ method. Sequences of primers used in this study are listed in Table 1. RT-qPCR assays were performed in duplicates for each cDNA and each primer couple and the experiment repeated two times.

**Table 1:** **List and sequences of primers used for RT-qPCR experiments**.

Primer name	5′ → 3′ Oriented sequence
DrActin For	CCCAGACATCAGGGAGTGAT
DrActin Rev	CACAATACCGTGCTCAATGG
DrHPRT1 For	CAGCGATGAGGAGCAAGGTTATG
DrHPRT1 Rev	GTCCATGATGAGCCCGTGAGG
DrIL1B For	GTCCACGTATGCGTCGCCCA
DrIL1B Rev	GGGGCAACAGGCCAGGTACA
DrTNFa For	GTGCAATCCGCTCAATCTGCACG
DrTNFa Rev	AATGGAAGGCAGCGCCGAGG

*Dr, Danio rerio; For, forward; Rev, reverse*.

### Statistical Analysis

Data obtained were expressed as mean ± SEM. Statistical differences among treatments were evaluated by Kruskall–Wallis method. *Post hoc* tests were used to identify statistical groups as described in each figure legend.

## Results

Manufacturer’s instructions indicate that Stifenia has to be solubilized in water. However, Stifenia is neither fully soluble in water nor in other classical solvents such as 100% DMSO, acetone 60% in water (v/v), ethanol 100%, and RPMI medium (data not shown), because it is composed of crushed fenugreek seeds. To study its effect on human PBMCs or zebrafish larvae, we used an aqueous soluble extract obtained as described in Section “Materials and Methods.” According to ANSES, the recommended use-concentration of Stifenia is 0.15–0.5% (m/v), which correspond to 1.5–5 mg mL^−1^ (ANSES 2012-1685, ANSES 2013-0227). In our study, we tested concentrations of Stifenia below these recommended use-concentrations.

### Stifenia Induces a Dose-Dependent Release of IL-1β in the Culture Medium

**Figure 1:**
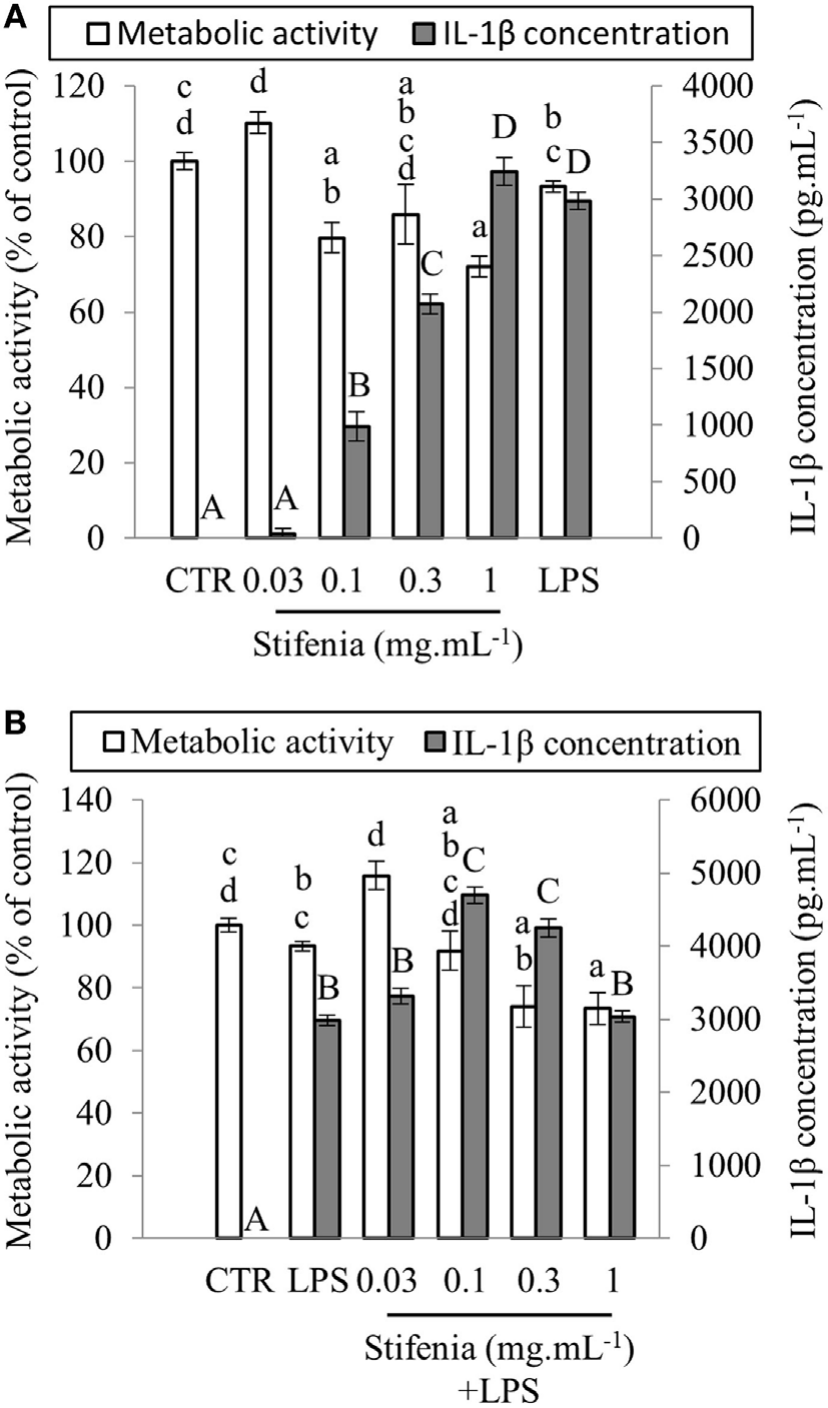
**Effect of Stifenia on human peripheral blood mononuclear cell (PBMC) metabolic activity (MA) and IL–1β production**. PBMCs were stimulated by different concentrations of Stifenia added in the culture medium. Cell MA (white bars) was estimated by the XTT assay and IL-1β (gray bars) was measured in the culture medium. **(A)** PBMCs were exposed to Stifenia for 20 h. **(B)** PBMCs were stimulated by addition of 10 ng mL^−1^ of LPS 30 min after the beginning of Stifenia exposure. Results are obtained from the blood donor CHR026_91. Bars represent the mean of eight technical replicates. Different letters (lowercase for the XTT assay, capitals for IL-1β) indicate statistical differences between groups (*p* < 0.05). If two conditions share one or several letters, there are no statistical differences. Statistical differences were determined using a Kruskal–Wallis test followed by a comparison with the Steel–Dwass–Critchlow–Fligner method. CTR, control non-treated cells.

Different concentrations of Stifenia (0.03–1 mg mL^−1^) were independently tested for 20 h on PBMC from nine different healthy human blood donors (Figure 1A; Figures S1 and S2 in Supplementary Material; Table 2). Cell MA was then measured using the XTT assay and IL-1β production was quantified in the culture medium using ELISA. A decrease of MA was observed from 0.1–1 mg mL^−1^ of Stifenia (Figure 1A). This decrease was observed with eight out of nine blood donors but to a different extend (Figure S1 in Supplementary Material). For these blood donors, it ranged from 9.5 to 33.2% when 0.3 mg mL^−1^ of Stifenia is used (Table 2).

**Table 2:** **Comparison of Stifenia or LPS exposure on metabolic activity (MA) and induced IL–1β production of peripheral blood mononuclear cells from nine different healthy human blood donors**.

Blood donor	CTR		Stifenia 0.3 mg mL^−1^		LPS	
	MA	IL-1β (pg mL^−1^)	MA	IL-1β (pg mL^−1^)	MA	IL-1β (pg mL^−1^)
CHR026_62	100.00 ± 0.51	3.68 ± 1.24	83.18 ± 3.39	228.35 ± 12.99	97.15 ± 1.33	276.53 ± 8.98
CHR026_76	100.00 ± 1.67	0.00 ± 0.00	87.22 ± 4.13	563.99 ± 29.62	127.17 ± 3.12	517.39 ± 42.65
CHR026_91	100.00 ± 2.25	0.00 ± 0.00	85.86 ± 7.90	2,069.41 ± 86.84	93.34 ± 1.46	2,984.13 ± 75.03
CHR026_93	100.00 ± 3.27	4.44 ± 2.06	74.68 ± 5.45	2,276.04 ± 224.08	99.61 ± 1.08	1,403.12 ± 26.94
CHR026_95	100.00 ± 2.70	2.13 ± 2.13	76.47 ± 3.31	3,268.65 ± 62.54	90.23 ± 2.98	1,631.87 ± 52.96
CHR026_128	100.00 ± 2.41	3.90 ± 3.90	90.57 ± 3.39	5.09 ± 2.96	102.30 ± 2.39	718.22 ± 28.63
CHR026_135	100.00 ± 9.91	0.00 ± 0.00	69.71 ± 7.14	715.86 ± 47.95	108.68 ± 2.10	76.13 ± 7.72
CHR026_152	100.00 ± 10.37	0.00 ± 0.00	66.8 ± 1.82	788.61 ± 423.84	90.65 ± 11.20	18.24 ± 18.24
CHR026_153	100.00 ± 1.41	0.00 ± 0.00	110.34 ± 3.36	946.77 ± 84.61	119.91 ± 2.28	626.87 ± 13.94

*Cell MA was estimated by the XTT assay, and IL-1β was measured in the culture medium 20 h after the addition of Stifenia or LPS. Results represent the mean between eight technical replicates*.

*CTR control non-treated cells*.

In culture medium from unexposed cells, IL-1β was either not detected or very close to background level (Figure 1A). In this cell batch CHR026_91, the lowest Stifenia concentration used (0.03 mg mL^–1^) did not induce IL-1β production. Higher concentrations progressively increased IL-1β release, which peaked at 3,240 pg mL^–1^ with 1 mg mL^–1^ of Stifenia. This quantity is equivalent to the one induced by 10 ng mL^–1^ of LPS. Although the pattern of IL-1β induction was always similar among the different PBMC batches tested, we noticed great variations in the quantities of IL-1β released (Table 2; Figure S1 in Supplementary Material). Thus, the lowest quantity found was 5 pg mL^–1^ after treatment with 0.3 mg mL^–1^ of Stifenia while the highest was 3,268 pg mL^–1^ (Table 2). The LPS-induced IL-1β production was also systematically measured and was also variable among the different blood donors. LPS (10 ng mL^–1^) induced the release of 18–2,984 pg mL^–1^ of IL-1β in the culture media, depending on the donor. No clear relationship was found between LPS- and Stifenia-induced IL-1β productions (Table 2; Figure S1 in Supplementary Material). In an exploratory experiment, we evaluated the production of others cytokines in the culture medium of Stifenia-exposed PBMC using a Multiplex assay (Table S1 in Supplementary Material). As observed with ELISA, Stifenia induced a dose-dependent production of IL-1β. We also detected a slight induction of TNFα and of the anti-inflammatory cytokine IL-10. No modulation of IFNγ, IL2, or IL-12p70 concentrations was observed.

### Stifenia Does Not Inhibit LPS-Induced Inflammation in PBMC

Literature frequently reported anti-inflammatory effects of fenugreek seed extracts (13, 25, 26). We thus tested the hypothesis that Stifenia could decrease the LPS-induced IL-1β production in PBMC. Figure 1B shows representative results of experiments conducted on PBMC isolated from six different blood donors. Cells were exposed with Stifenia as previously described and then stimulated 30 min later with 10 ng mL^–1^ LPS. IL-1β was measured 20 h later. Stifenia pretreatment did not reduce LPS-induced IL-1β production (Figure 1B; Figure S2 in Supplementary Material). For each Stifenia concentration tested, the IL-1β produced was either higher or equal to the LPS alone-induced IL-1β synthesis. Regarding MA, while LPS alone never reduced it, Stifenia pre-exposure (0.3–1 mg mL^–1^) decreased the MA in a dose-dependent manner as observed when used alone (Figure 1B; Figure S2 in Supplementary Material).

### Stifenia Does Not Contain Contaminating Microorganisms

**Figure 2:**
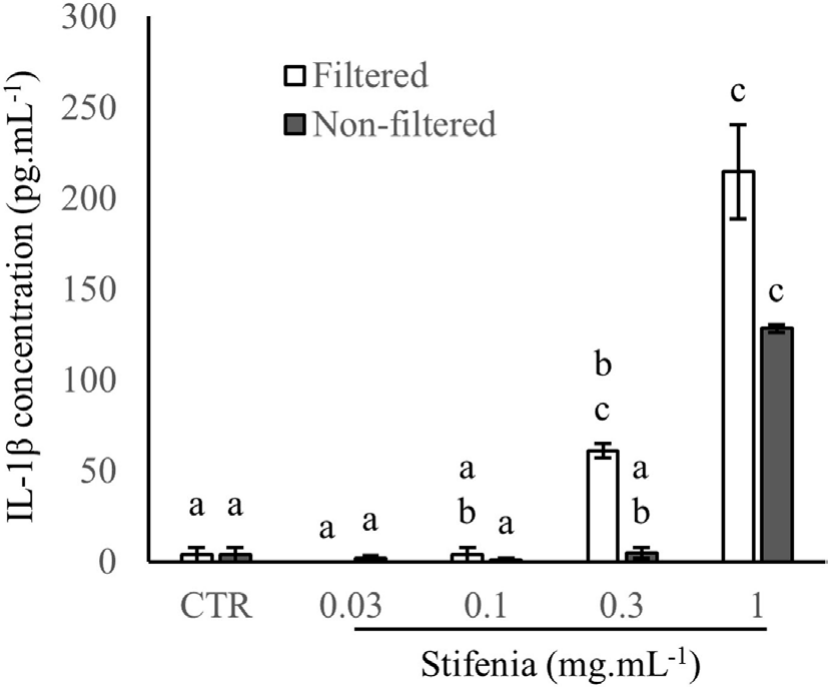
**Comparison of filtrated and non-filtrated Stifenia preparation on IL-1β production**. Results are obtained from the blood donor CHR026_128. IL-1β was measured in the culture medium 20 h after the addition of filtered (white bars) and non-filtered (gray bars) Stifenia. Each bar corresponds to four technical replicates. Statistical differences were determined using a Kruskal–Wallis test followed by a comparison with the Conover–Iman method. Similar letters indicate no significant differences (*p* < 0.05). CTR, control non-treated cells. This experiment was repeated once with peripheral blood mononuclear cell from another blood donor (CHR026_135) with similar results (data not shown).

In order to check if inflammatory activity of Stifenia was not due to contamination by microorganisms, we compared results obtained from a Stifenia preparation that was either directly used for cell treatment or previously filtered on 0.22 µm pore size membrane. In both cases, a dose-dependent induction of the cytokine production was found. IL-1β induction was not significantly different between filtered and non-filtered Stifenia although it seems that filtered Stifenia induce more IL-1β (Figure 2).

### Inflammatory Effect of Stifenia Is Inhibited by Caspase Inhibitor

**Figure 3:**
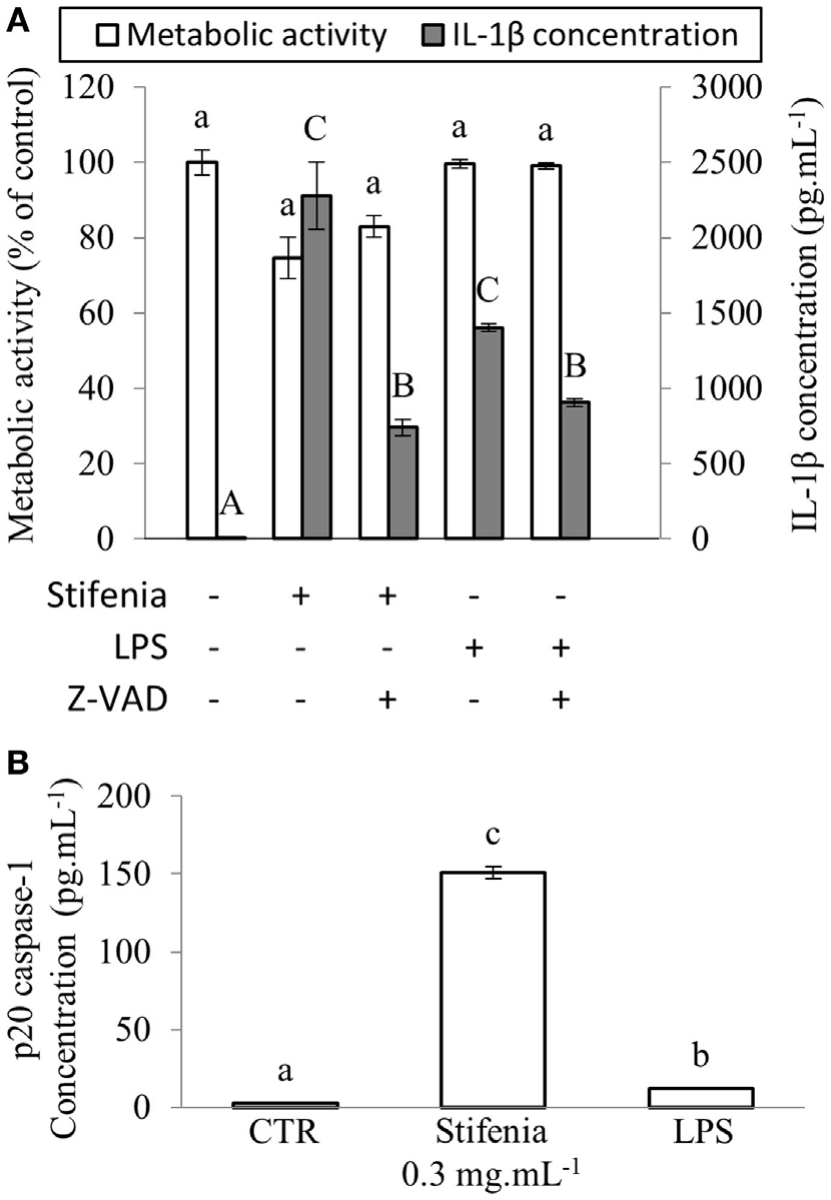
**Stifenia-induced IL-1β production is mediated by caspase1**. **(A)** Peripheral blood mononuclear cells were stimulated by 0.3 mg mL^−1^ of Stifenia or 10 ng mL^−1^ of LPS. Cell metabolic activity (MA) (white bars) was estimated by the XTT assay and IL-1β (gray bars) was measured in the culture medium 20 h after treatment. Five micromolars Z-VAD-FMK (Z-VAD) were added simultaneously to Stifenia in the culture medium. LPS was added 30 min after Z-VAD-FMK treatment. The results showed are obtained from the blood donor CHR026_93. Bars represent the mean between eight technical replicates. Lowercase and capital letters indicate statistical groups for MA and IL-1β concentration, respectively. Different letters indicate statistical differences between groups (*p* < 0.05). If two conditions share one or several letters, there are no statistical differences. CTR, control non-treated cells. **(B)** Quantity of p-20 Caspase-1 fragment was measured using ELISA in the culture medium 20 h after the addition of 0.3 mg mL^−1^ of Stifenia or 10 ng mL^−1^ of LPS. Each bar corresponds to a mean of eight technical replicates. Results are obtained from the blood donor CHR026_93. This experiment was repeated once on another blood donor with similar results (data not shown). **(A,B)** Statistical differences were determined using a Kruskal–Wallis test followed by a comparison with the Steel–Dwass–Critchlow–Fligner method. Similar letters indicate no significant differences (*p* < 0.05). CTR, control non-treated cells.

IL-1β is processed to its active released form by caspase-1 through the inflammasome multiprotein complex NLRP3 (7, 27). We used a pan-inhibitor of caspases in order to test whether IL-1β released upon Stifenia stimulation was generated *via* an inflammasome complex. Sixty per cent of the cytokine production induced by Stifenia was abolished by Z-VAD-FMK. As expected, Z-VAD-FMK also strongly reduced LPS-induced IL-1β production (Figure 3A; Figure S3 in Supplementary Material). We then checked the involvement of caspase-1 by ELISA on the production of IL-1β induced by Stifenia. In unexposed cells, detection of p-20 caspase-1 fragment in the culture medium was close to the detection threshold. Stifenia 0.3 mg mL^−1^ induced a huge release of extracellular p-20 Caspase-1 fragment that is much higher than with LPS exposure alone (Figure 3B).

### Stifenia Induces Cell Death of PBMC

Using flow cytometry and PI, we checked if a 20-h Stifenia exposure can induce PBMC cell death (Figure S4 in Supplementary Material). In our experiments performed on two independent blood donors, the lowest concentrations of Stifenia tested (0.03 and 0.1 mg mL^−1^) did not induced cell death. However, treatment of PBMC with 0.3 or 1 mg mL^−1^ induced cell death that ranged from 22.8 to 38.6% depending of the blood donor. LPS (10 ng mL^−1^) alone did not significantly induced cell death as described by others. Furthermore, LPS treatment did not modify or potentiate the toxicity of Stifenia.

### Stifenia Induces Cytokine Gene Expression in Zebrafish Larvae

**Figure 4:**
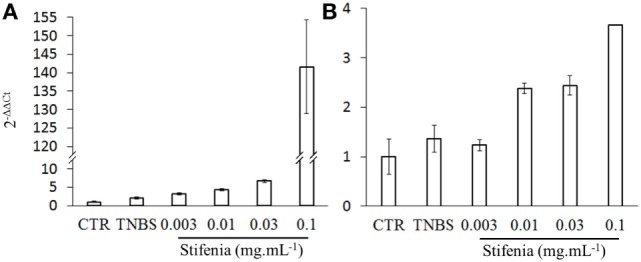
**Effect of Stifenia treatment on cytokine gene expression in zebrafish larvae**. Induction of cytokine gene expression in zebrafish larvae exposed for 20 h to Stifenia aqueous extract. The transcript levels of IL-1β **(A)** and TNFα **(B)** were quantified by RT-qPCR using the *Danio rerio* HPRT1 gene as housekeeping gene and normalized to the transcript level of larvae that were not exposed (CTR) using 2^−ΔΔCt^ method. Data are the means of duplicates from one representative experiment out of two.

To analyze the inflammatory effect of Stifenia on a whole organism, we used zebrafish larvae. Using RT-qPCR, we investigated the effect of Stifenia on IL-1β and TNFα gene expression. Twelve to fifteen zebrafish larvae were exposed to different concentrations of Stifenia for 20 h and then harvested for total RNA extraction. TNBS used as a reference inflammatory compound induced a slight IL-1β gene expression compared to unexposed fishes as previously described (24, 28). The lowest concentration of Stifenia induced a higher IL-1β gene expression than TNBS. Furthermore, we observed a dose-dependent effect of Stifenia on IL-1β gene expression. For TNFα gene expression, an increase was observed from 0.01 mg mL^−1^ of Stifenia exposure (Figure 4).

## Discussion

### Inflammatory Effect of Stifenia

In this study, we demonstrated that the commercial plant protection product Stifenia decreased the MA of PBMC and affected cell viability. Stifenia also induced IL-1β production in all the PBMC batches tested, but to a different extent depending on the blood donor. IL-1β is an inflammatory cytokine circulating at very low levels (0.5–10 pg mL^−1^) in blood stream of healthy people (29, 30). In our culture conditions, unstimulated PBMCs produce similar concentration of IL-1β in the culture medium. When PBMCs were exposed to Stifenia 0.1 mg mL^−1^, they released around 500 pg mL^−1^ of IL-1β, which corresponds to a 50- to 1,000-fold increase of IL-1β concentration compared either to control conditions or to the concentrations of IL-1β found in blood of healthy people. It could be hypothesized that such an increase of IL–1β concentration induced by Stifenia could have physiological effects.

The inflammatory effect of Stifenia contrasts with other published data indicating that fenugreek seed extracts exhibit anti-inflammatory properties [reviewed by Goyal et al. (13)]. We did not detect any anti-inflammatory properties when Stifenia was used as pretreatment before LPS stimulation. A similar protocol was, however, efficient when used with other natural or synthetic compounds (31, 32). The discrepancy between our data and published ones could be explained. First, we used an aqueous extract of Stifenia while other studies used organic extracts. For instance, Mandegary et al. (25) performed a methanol extraction of fenugreek seeds followed by a fractionation using water and different organic solvents. They showed that the aqueous fraction mostly contains flavonoids and inhibits carrageenan-induced paw edema in mice. Furthermore, ethanol extract of fenugreek seeds reduced Freund’s complete adjuvant-induced arthritis in rats by lowering cytokine induction (26). We suspect that the anti-inflammatory effect found in these extracts (25, 26) could be hidden or lowered by other components present in our aqueous Stifenia extract. Second, the biological/experimental models used to demonstrate the anti-inflammatory effects were different from the human PBMC model used in this study. However, the use of an aqueous extract in our study is of relevance because it reflects what is done by farmers when Stifenia is prepared according to the manufacturer’s instructions. In other words, the aqueous extract we tested is similar to what is sprayed by farmers on crops.

We showed that caspase-1 was involved in the inflammatory response induced by Stifenia suggesting a role for the inflammasome NLRP3. IL-1β is produced by immune cells as a response to NLRP3 inflammasome activation when cells are confronted to PAMP but also to different danger signals (DAMP) of metabolic origin (27, 33). It could be suspected that some compounds contained in the Stifenia extract could stimulate the NLRP3 inflammasome and lead to caspase-1 activation that is responsible for pro-IL-1β processing.

Because Stifenia induced IL-1β production in a caspase-1-dependant pathway, one may suggest that Stifenia effects are partly due to a contamination with LPS which induce pro-IL-1β gene transcription after its perception by TLR4, a phenomenon known as priming. Thus, the presence of LPS in Stifenia aqueous extract has to be considered. To test this hypothesis, we checked for the presence of 3OH-fatty acids that compose LPS in the Stifenia aqueous extract using an HPLC/MSMS method (34). Interestingly, we detected 3OH-C10:0, 3OH-C12:0, 3OH-C14:0, and 3OH-C16:0 revealing a LPS contamination of Stifenia (data not shown). After filtration of the Stifenia aqueous extract on a poly-lysine column, LPS contamination was decreased by about 40%. However, this extract was still efficient in inducing IL-1β production on PBMC (data not shown). Furthermore, PBMC harvested from two blood donors, CHR026_135, and CHR026_152 (Figures S1D,E in Supplementary Material), are not or very slightly susceptible to LPS while they are highly reactive to Stifenia. We also showed that Stifenia exposure induced cell death of PBMC, whereas LPS did not. All these data suggest that Stifenia-induced IL-1β production and Stifenia-induced cell death are unlikely due to a direct effect of LPS, but we cannot rule out that LPS present in Stifenia aqueous extract could prime pro- IL-1β gene transcription.

### Are Inflammatory Effects Relevant for Human Health?

The ANSES has assessed the acceptable exposure level of Stifenia for operators (0.3–2.9 mg kg^−1^ of body weight/day), workers (3–9 mg kg^−1^ of body weight/day), and neighborhood (0.01–0.09 mg kg^−1^ of body weight/5 min of continuous exposition) (ANSES 2012-1685 and 2013-0227). Even if these estimated contaminating doses seems to be low, recurrent or longer exposures have not been tested so far. Our results demonstrated some inflammatory properties and toxicity of Stifenia on blood mononuclear cells. Of importance, the potential toxicity of fenugreek was recently highlighted by some studies both in human and animals. Thus, a survey in Moroccan maternity hospital has linked consumption of fenugreek by pregnant women to congenital malformations (14, 35). In mice, feeding females during the entire period of pregnancy with a lyophilized aqueous extract from fenugreek seeds affects their reproduction and shows teratogenic and foetotoxic effects (4). Khalki et al. (15) also reported growth retardation and altered neurobehavioral performance of mice prenatally exposed to fenugreek seed extracts. Antifertility effects of fenugreek seed extract has also been reported in rabbits (17), and other toxic effects have been described in mice, rats and rabbits (16, 18). However, since fenugreek seeds have been used for centuries in traditional medicine or in food, many tests were not included in the risk assessment of Stifenia (36). Our results pointed out the possible danger of an extensive use of the plant defense stimulator Stifenia at the level of human health.

### Environmental Toxicity

Our data demonstrated *in vivo* inflammatory effect of Stifenia on zebrafish larvae. We observed an induction of IL-1β and TNF-α gene expression that started at Stifenia concentration as low as 3 and 10 µg mL^−1^, respectively. The predicted environmental concentration (PEC) of Stifenia in surface water was established by the ANSES at 0.160 µg mL^–1^ (ANSES 2012-1685). This is only 20-fold lower than the 3 µg mL^−1^ concentration that induced significant IL-1β gene expression in our experiments. This raises the issue of whether a longer exposure of zebrafish larvae to the PEC concentration could have similar inflammatory effects to those we observed. However, the methods used to predict PEC in surface water are controversial and could not reflect the actual concentrations of the molecule in the environment (37). Recently, it has been shown that the PEC calculated for several insecticides are underestimated by the procedure used by the European Union (38). Regarding the toxicity of fenugreek on animals, a study showed that topical application of 6 µg of fenugreek acetonic seed extract on two coleopteran species, *Acanthoscelides obtectus* and *Tribolium castaneum*, decreased their fertility and induced their mortality. These authors also showed that the presence of fenugreek seeds in the immediate environment of insects was sufficient to kill them (19). All these results suggest that the use of Stifenia could have adverse effects on non-target organisms.

## Conclusion

Our results demonstrated unexpected effects of a plant protection product on human and animal health. As written by Burketova et al. (2), “although bio-based products are of natural origin, direct toxicity of these products to human, animals, insect, microbe communities, or even plants must be studied carefully to avoid toxicity as observed with classical pesticides.” The human cell-based approach developed in this work revealed a high sensitivity concerning inflammatory properties of a plant protection product. These tests could be routinely used to screen the potential adverse effects of this type of compounds that can potentially cross-react with human innate immunity. This should be the first step before engaging more expensive studies on animal models and according to the European legislation, and this approach fits with the goal of reducing studies on animal models (No. 1107/2009).

## Ethics Statement

For Zebrafish larvae, there is no need of ethics committee because they were euthanized at 120-h postfertilization [EU Directive 2010/63/EU, Strähle et al. (21) and Geisler et al. (22)]. For human PBMC, there is no need of ethics committee. PBMCs were obtained from Etablissement Français du Sang (EFS) according to agreement No. DECO-14-0124 and EFS do not indicate any information on PBMC donors.

## Author Contributions

LT, OL, and JLC designed this work. LT, JCo, OL, and JLC performed most of the experiments. JCh supervised zebrafish experiments. LT, JCo, SD, JCh, DW, OL, and JLC contributed substantially to the completion of this work. LT, OL, and JLC wrote the manuscript. All the authors read and approved the final manuscript.

## Conflict of Interest Statement

The authors declare that the research was conducted in the absence of any commercial or financial relationships that could be construed as a potential conflict of interest.

## Supplementary Material

Table S1 **Cytokine concentrations in culture medium of Stifenia-exposed peripheral blood mononuclear cells**. Cytokines were measured in the culture medium 20 h after the addition of Stifenia at the indicated concentrations using a multiplex kit (ProcartaPlex human simplex, Affymetrix, France) according to the manufacturer’s instructions on blood donor CHR026_91. C, concentration; IF, induction factor; CTR, control non-treated cells.

Figure S1 **Effect of Stifenia IL-1β production and metabolic activity (MA)**. Peripheral blood mononuclear cells were exposed to different concentrations of Stifenia added in the culture medium for 20 h. Cell MA (white bars) was estimated by the XTT assay, and IL-1β (gray bars) was measured in the culture medium. Results are obtained from six blood donors (**A**–**F**). Bars represent the mean of eight technical replicates. Different letters (lowercase for the XTT assay, capitals for IL-1β) indicate statistical differences between groups (*p* < 0.05). Statistical differences were determined using a Kruskal–Wallis test followed by a comparison with the Steel–Dwass–Critchlow–Fligner method. CTR, control non-treated cells.

Figure S2 **Effect of Stifenia on LPS-induced IL-1β production and metabolic activity (MA)**. Peripheral blood mononuclear cells were stimulated by addition of 10 ng mL^−1^ of LPS 30 min after the beginning of Stifenia exposure. Cell MA (white bars) was estimated by the XTT assay, and IL-1β (gray bars) was measured in the culture medium. Results are obtained from five blood donors (**A**–**E**). Bars represent the mean of eight technical replicates. Different letters (lowercase for the XTT assay, capitals for IL-1β) indicate statistical differences between groups (*p* < 0.05). Statistical differences were determined using a Kruskal–Wallis test followed by a comparison with the Steel–Dwass–Critchlow–Fligner method. CTR, control non-treated cells.

Figure S3 **Effect of ZVAD on Stifenia- or LPS-induced IL-1β production and metabolic activity (MA)**. Cell MA (white bars) was estimated by the XTT assay, and IL-1β (gray bars) was measured in the culture medium 20 h after treatment. Five micromolars Z-VAD-FMK (Z-VAD) were added simultaneously to Stifenia (0.3 mg mL^−1^) in the culture medium or 30 min before the addition of LPS (10 ng mL^−1^). Results are obtained from blood donors CHR026_95 (A) and CHR026_153 (B). Bars represent the mean between eight technical replicates. Different letters (lowercase for the XTT assay, capitals for IL-1β) indicate statistical differences between groups (*p* < 0.05). Statistical differences were determined using a Kruskal–Wallis test followed by a comparison with the Steel–Dwass–Critchlow–Fligner method. CTR, control non-treated cells.

Figure S4 **Peripheral blood mononuclear cell (PBMC) viability after Stifenia treatment**. Cell viability was estimated by propidium iodide (PI) staining 20 h after PBMC treatments. The results shown are obtained from blood donors CHR026_153 **(A,B)** and CHR026_152 **(C,D)**. **(A,C)** Cell death was expressed as a percentage of PI-stained cells vs. total cells. Bars represent the mean between four technical replicates. Different letters indicate statistical differences between groups (*p* < 0.05). Statistical differences were determined using a Kruskal–Wallis test followed by a comparison with the Dunn’s method. **(B,D)** Flow cytometry diagram of PI-stained cells 20 h after treatment. For each treatment, a representative diagram out of four technical replicates is shown for the blood donor CHR026_153 **(B)** or the blood donor CHR026_152 **(D)**.
