# Supplementary material for: Inflammatory Effects of the Plant Protection Product Stifenia (FEN560) on Vertebrates
Source: Front Public Health. 2017 Apr 24;5:74. doi: 10.3389/fpubh.2017.00074 (PMC5402227; doi:10.3389/fpubh.2017.00074)
Supplement: Table S1 — Cytokine concentrations in culture medium of Stifenia-exposed peripheral blood mononuclear cells. Cytokines were measured in the culture medium 20 h after the addition of Stifenia at the indicated concentrations using a multiplex kit (ProcartaPlex human simplex, Affymetrix, France) according to the manufacturer’s instructions on blood donor CHR026_91. C, concentration; IF, induction factor; CTR, control non-treated cells. [file Table_1.PDF]

	IL-1beta		TNF-α		IFN-γ		IL-2		IL-10		IL-12	
	C	IF	C	IF	C	IF	C	IF	C	IF	C	IF
CTR	3.00	1.00	3.00	1.00	0.50	1.00	16.00	1.00	1.00	1.00	0.60	1.00
Stifenia 0,1 $mg.mL^{-1}$	556.63	185.54	14.82	4.94	0.70	1.40	13.00	0.81	5.19	5.19	0.40	0.67
Stifenia 0,3 $mg.mL^{-1}$	1374.17	458.06	81.85	27.28	0.80	1.60	16.00	1.00	23.26	23.26	0.50	0.83

Teyssier at al. Supp. Tab. 1
