# Supplementary figures and images for: Inflammatory Effects of the Plant Protection Product Stifenia (FEN560) on Vertebrates
Source: Front Public Health. 2017 Apr 24;5:74. doi: 10.3389/fpubh.2017.00074 (PMC5402227; doi:10.3389/fpubh.2017.00074)

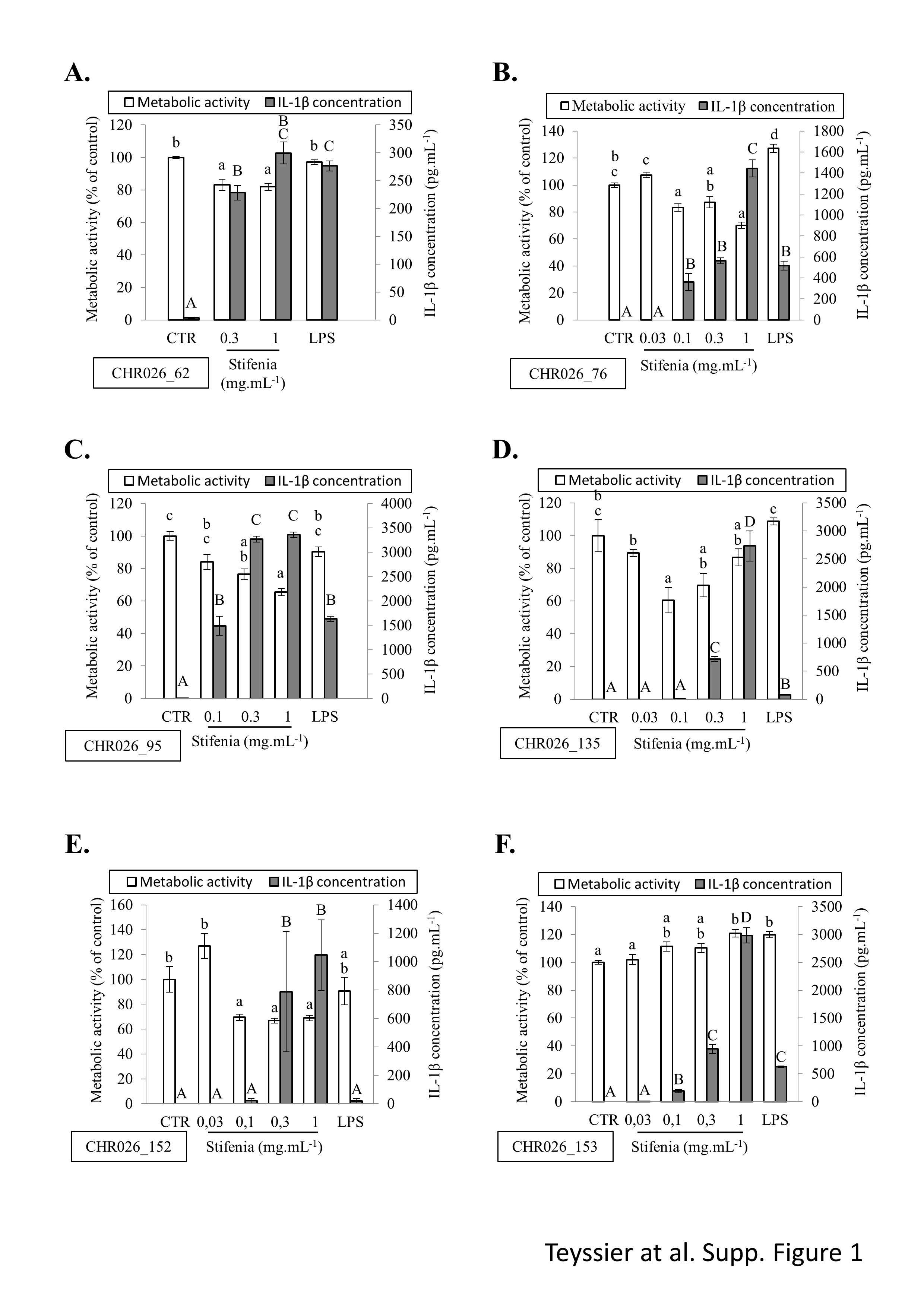

Supplement: Figure S1 — Effect of Stifenia IL-1β production and metabolic activity (MA). Peripheral blood mononuclear cells were exposed to different concentrations of Stifenia added in the culture medium for 20 h. Cell MA (white bars) was estimated by the XTT assay, and IL-1β (gray bars) was measured in the culture medium. Results are obtained from six blood donors (A–F). Bars represent the mean of eight technical replicates. Different letters (lowercase for the XTT assay, capitals for IL-1β) indicate statistical differences between groups (p < 0.05). Statistical differences were determined using a Kruskal–Wallis test followed by a comparison with the Steel–Dwass–Critchlow–Fligner method. CTR, control non-treated cells. [file Image_1.TIF]

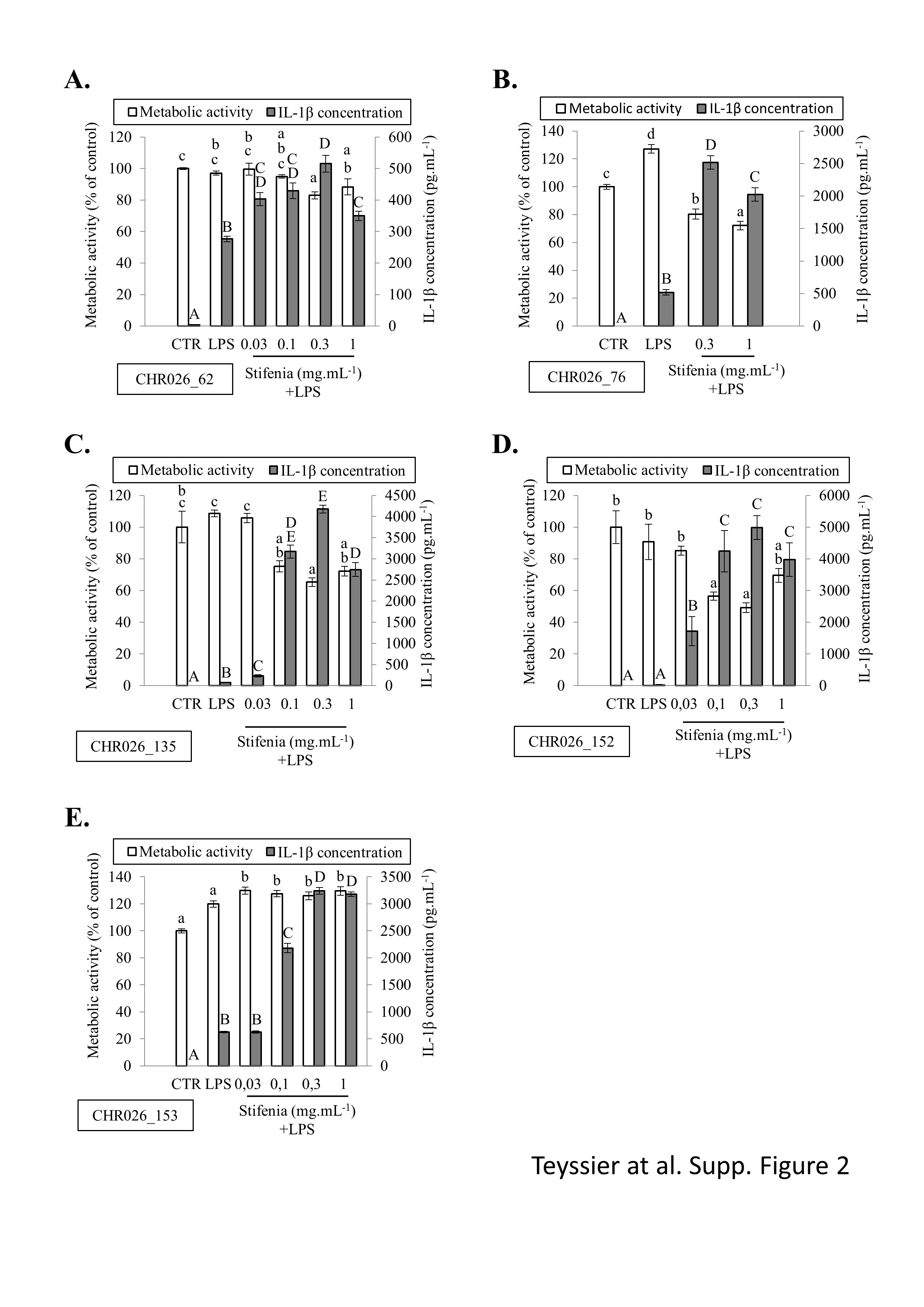

Supplement: Figure S2 — Effect of Stifenia on LPS-induced IL-1β production and metabolic activity (MA). Peripheral blood mononuclear cells were stimulated by addition of 10 ng mL−1 of LPS 30 min after the beginning of Stifenia exposure. Cell MA (white bars) was estimated by the XTT assay, and IL-1β (gray bars) was measured in the culture medium. Results are obtained from five blood donors (A–E). Bars represent the mean of eight technical replicates. Different letters (lowercase for the XTT assay, capitals for IL-1β) indicate statistical differences between groups (p < 0.05). Statistical differences were determined using a Kruskal–Wallis test followed by a comparison with the Steel–Dwass–Critchlow–Fligner method. CTR, control non-treated cells. [file Image_2.TIF]

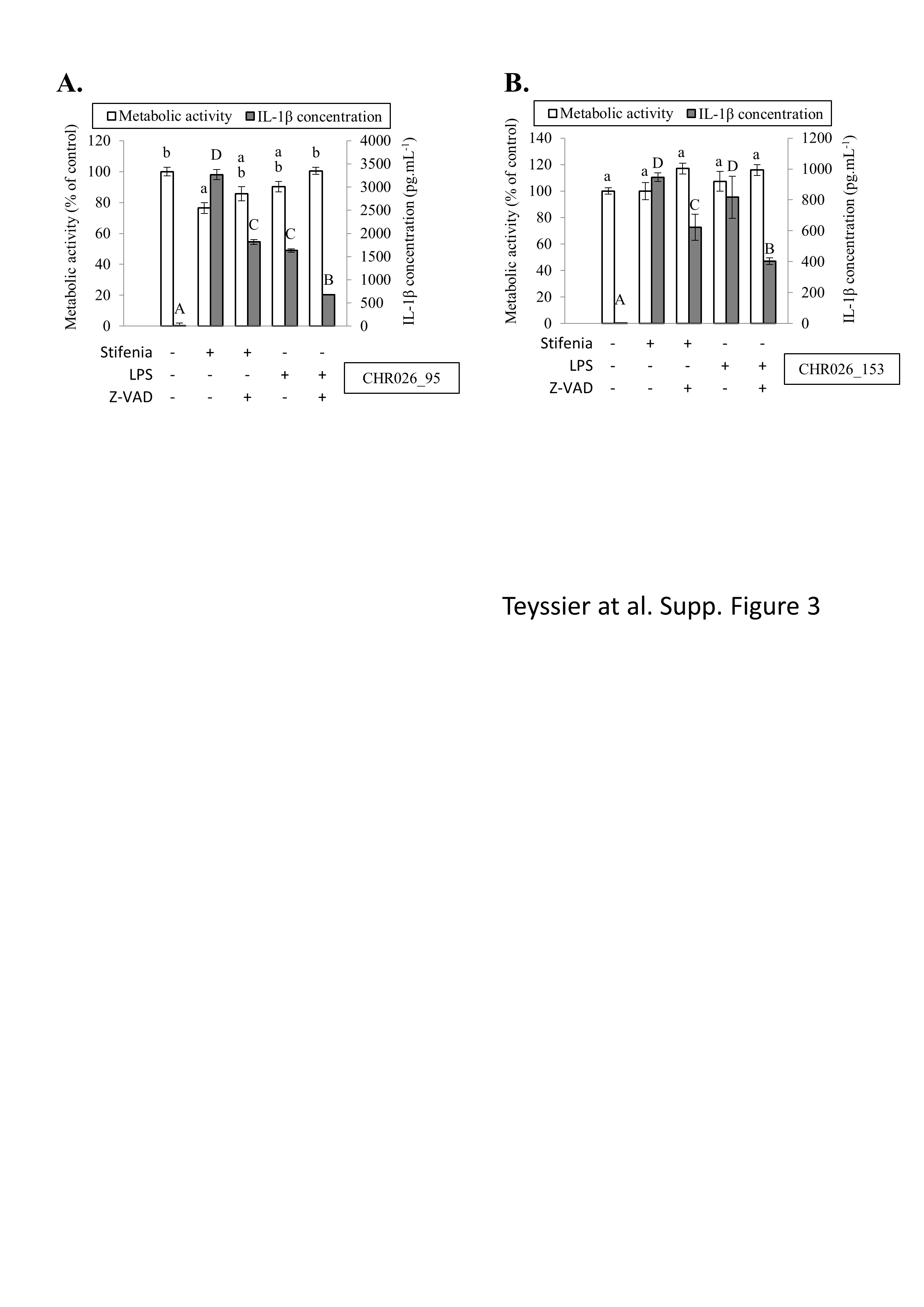

Supplement: Figure S3 — Effect of ZVAD on Stifenia- or LPS-induced IL-1β production and metabolic activity (MA). Cell MA (white bars) was estimated by the XTT assay, and IL-1β (gray bars) was measured in the culture medium 20 h after treatment. Five micromolars Z-VAD-FMK (Z-VAD) were added simultaneously to Stifenia (0.3 mg mL−1) in the culture medium or 30 min before the addition of LPS (10 ng mL−1). Results are obtained from blood donors CHR026_95 (A) and CHR026_153 (B). Bars represent the mean between eight technical replicates. Different letters (lowercase for the XTT assay, capitals for IL-1β) indicate statistical differences between groups (p < 0.05). Statistical differences were determined using a Kruskal–Wallis test followed by a comparison with the Steel–Dwass–Critchlow–Fligner method. CTR, control non-treated cells. [file Image_3.TIF]

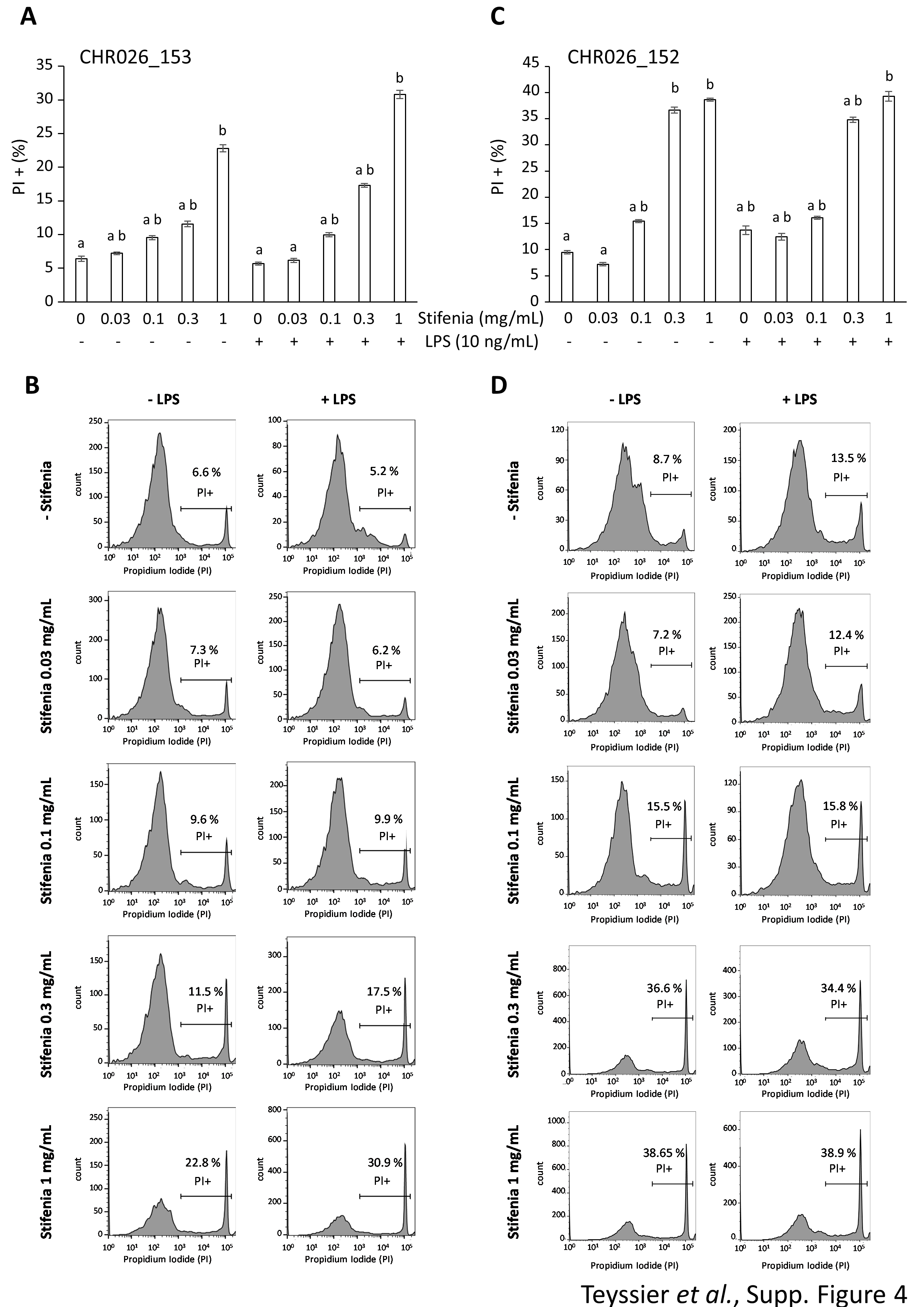

Supplement: Figure S4 — Peripheral blood mononuclear cell (PBMC) viability after Stifenia treatment. Cell viability was estimated by propidium iodide (PI) staining 20 h after PBMC treatments. The results shown are obtained from blood donors CHR026_153 (A,B) and CHR026_152 (C,D). (A,C) Cell death was expressed as a percentage of PI-stained cells vs. total cells. Bars represent the mean between four technical replicates. Different letters indicate statistical differences between groups (p < 0.05). Statistical differences were determined using a Kruskal–Wallis test followed by a comparison with the Dunn’s method. (B,D) Flow cytometry diagram of PI-stained cells 20 h after treatment. For each treatment, a representative diagram out of four technical replicates is shown for the blood donor CHR026_153 (B) or the blood donor CHR026_152 (D). [file Image_4.TIF]
